# Complete Genome Sequence of Serratia marcescens FY, Isolated from Drosophila melanogaster

**DOI:** 10.1128/MRA.00755-20

**Published:** 2020-11-05

**Authors:** Wei Liu, Ruxue Kang, Kah Leong Lim, Eng King Tan

**Affiliations:** a Department of Neurology, National Neuroscience Institute, Singapore, Singapore; b Department of Medical Laboratory Science, Shanxi Medical University Fenyang College, Shanxi, China; c Neuroscience and Behavioural Disorders Program, Duke-NUS Medical School, Singapore, Singapore; University of Maryland School of Medicine

## Abstract

In nature, the fruit fly frequently encounters various pathogens that cause a decrease in host fitness. Here, we present the genome sequence of Serratia marcescens strain FY, which was isolated from the intestines of a *Drosophila* specimen. The complete genome sequence comprises one chromosome of 5,074,453 bp and two plasmids of 100,934 bp and 87,789 bp. A total of 4,891 coding sequences are predicted from this assembly.

## Abstract (precis)

In nature, the fruit fly frequently encounters various pathogens that cause a decrease in host fitness. Here, we present the genome sequence of Serratia marcescens strain FY that was isolated from the intestines of a *Drosophila* sp. specimen. The complete genome sequence comprises one chromosome of 5,074,453 bp and two plasmids of 100,934 bp and 87,789 bp. A total of 4,891 coding sequences are predicted from this assembly.

## ANNOUNCEMENT

Serratia marcescens is an opportunistic, Gram-negative bacterium belonging to the family *Enterobacteriaceae* (1). S. marcescens has been reported to infect other animals, including some insects (2, 3). S. marcescens was selected as a model to investigate how the fruit fly, Drosophila melanogaster, handles invasive pathogens (4 – 6). Here, we report the complete genome sequence of S. marcescens FY, isolated from D. melanogaster.

This isolate was recovered from the midguts of D. melanogaster specimens captured in the orchard (lat 37.265454, long 111.787759). The diluted lysate of homogenized guts was plated onto LB agar medium, and the medium was incubated for 2 days under aerobic conditions at 28°C. Single colonies were serially streaked twice for purification, and an individual colony was finally picked into LB broth for growth and stored in LB broth with 15% glycerol at −20°C. Total DNA was extracted using the DNeasy PowerLyzer PowerSoil kit (Qiagen) (7). The quantity of DNA was examined with a NanoDrop 2000 instrument, and the DNA was sent to Beijing Novogene Bioinformatics Technology Co., Ltd. (Beijing, China). Genomic DNA was sheared into the desired fragment size using a g-TUBE device (Covaris). Library preparation was performed with a SMRTbell template kit v3.0 (Pacific Biosciences). Fragments of the template smaller than 20 kbp were removed using the BluePippin size selection system v10 (Sage Science), and the constructed library was validated using a Bioanalyzer 2100 instrument (Agilent Technologies). The SMRTbell library was sequenced using PacBio single-molecule real-time (SMRT) sequencing with C4 chemistry on the PacBio RS II platform. SMRT Analysis v2.3.0 (Pacific Biosciences) was used to perform demultiplexing, base calling, and quality filtering of the raw read sequences, and *de novo* assembly was conducted according to the RS Hierarchical Genome Assembly Process (HGAP) v3.0 workflow (8). Subsequently, Circlator v1.5.5 was used to ensure circularization of the chromosome by trimming overlapping ends and rotating to the start of the *dnaA* gene (9). After filtering 151,653 subreads, we retained a pool of 1,427,584,714 bp from 151,212 long reads with an *N*_50_ value of 10,621 bp, yielding 223-fold genome coverage. Gene predictions and annotations were provided by NCBI using the NCBI Prokaryotic Genome Annotation Pipeline (10). The SEED subsystem was used via Rapid Annotations using Subsystems Technology (RAST) for functional categorization of the predicted proteins (11). Default software parameters were used except where otherwise noted.

The complete genome of S. marcescens FY contains one circular chromosome and two circular plasmids, named Plas1 and Plas2. The chromosomal genome assembly was 5,543,750 bp, with a G+C content of 59.7%. The genomic sequence from nucleotide positions 4618264 to 4619794 encodes 16S rRNAs and displays 99.8% (identities, 1,528/1,531) similarity with a published 16S rRNA gene sequence of S. marcescens (12). The plasmid assemblies were 100,934 bp (G+C content, 54.7%) and 87,789 bp (G+C content, 53.2%), respectively. The genome annotation was completed by submission to the NCBI Prokaryotic Genome Automatic Annotation Pipeline (PGAAP) to obtain a total of 4,891 predicted genes, 22 rRNAs, 89 tRNAs, and 22 noncoding RNAs. The S. marcescens FY genome reveals not only the cellular and catabolic adaptation mechanisms of this bacterium to *Drosophila* intestine but also the acquisition of multiple plasmids.

### Data availability.

The genome sequence and associated data for S. marcescens FY were deposited under GenBank accession no. CP053378 (chromosome), CP053379 (plasmid 1), and CP053380 (plasmid 2). The raw reads are available at the NCBI SRA (accession no. SRX8587283). The BioProject number is PRJNA631339, and the BioSample accession number is SAMN14859508.
